# Extracellular Vesicles Released by Human Induced-Pluripotent Stem Cell-Derived Cardiomyocytes Promote Angiogenesis

**DOI:** 10.3389/fphys.2018.01794

**Published:** 2018-12-14

**Authors:** Julie A. Dougherty, Naresh Kumar, Mohammad Noor, Mark G. Angelos, Mohsin Khan, Chun-An Chen, Mahmood Khan

**Affiliations:** ^1^Dorothy M. Davis Heart & Lung Research Institute, The Ohio State University Wexner Medical Center, Columbus, OH, United States; ^2^Department of Emergency Medicine, The Ohio State University Wexner Medical Center, Columbus, OH, United States; ^3^Center for Metabolic Disease Research, Lewis Katz School of Medicine, Temple University, Philadelphia, PA, United States; ^4^Department of Physiology and Cell Biology, The Ohio State University Wexner Medical Center, Columbus, OH, United States

**Keywords:** extracellular vesicles, stem cells, angiogenesis, cardiomyocytes, cardiac regeneration

## Abstract

Although cell survival post-transplantation is very low, emerging evidence using stem cell therapy for myocardial repair points toward a primary role of paracrine signaling mechanisms as the basis for improved cardiac function, decreased fibrosis, and increased angiogenesis. Recent studies have demonstrated that extracellular vesicles (EVs) such as exosomes secreted by stem cells stimulate angiogenesis, provide cytoprotection, and modulate apoptosis. However, the angiogenic potential of EVs secreted from human induced pluripotent stem cell-derived cardiomyocytes (hiPSC-CM), a terminally differentiated cell type, has not been elucidated yet. Therefore, the main objective of this study is to isolate, characterize, and evaluate the *in vitro* angiogenic potential of EVs collected from hiPSC-CM conditioned media. The hiPSC-CM were cultured for 2 weeks and EVs were isolated from cell culture medium. Isolated EVs were characterized by transmission electron microscopy (TEM), nanoparticle tracking analysis, and immunoblotting. Furthermore, the angiogenic potential of these EVs was evaluated by tube formation, wound-healing, and cell-proliferation assays in bovine aortic endothelial cells (BAEC). In addition, gene expression levels of growth factors was evaluated in hiPSC-derived endothelial cells (hiPSC-EC) treated with hiPSC-CM-derived EV (CM-EVs) to assess their role in promoting angiogenesis. TEM imaging of CM-EVs showed a presence of a double-membrane bound structure, which is a characteristic of EV. Nanoparticle tracking analysis further confirmed the size and shape of the secreted particles to be consistent with EVs. Furthermore, EV-specific markers (CD63 and HSP70) were enriched in these particles as illustrated by immunoblotting. Most importantly, BAEC treated with 100 μg/ml of CM-EVs showed significant increases in tube formation, wound closure, and cell proliferation as compared to control (no-EVs). Finally, treatment of hiPSC-EC with CM-EVs induced increased expression of pro-angiogenic growth factors by the endothelial cells. Overall, our results demonstrated that EVs isolated from hiPSC-CM enhance angiogenesis in endothelial cells. This acellular/cell-free approach constitutes a potential translational therapeutic to induce angiogenesis in patients with myocardial infarction.

## Introduction

Cardiovascular disease (CVD) is the leading cause of death in the United States, with coronary heart (CHD) accounting for a majority disease of the cardiovascular events in Americans under 75 years of age (Benjamin et al., 2017). Myocardial infarction (MI) occurs when blood flow to the heart is blocked, damaging heart muscle. Unfortunately, scar formation and maladaptive responses after MI lead to decreased left ventricular function and, ultimately, heart failure (Prabhu and Frangogiannis, 2016). On average, every 40 s an American suffers an MI, of which 21% are silent (Benjamin et al., 2017). Accordingly, treatment of MI is very costly with aggregate United States hospital costs for acute MI patients of $11.5 billion in 2011 (Pfuntner et al., 2006). Current pharmacological and surgical treatments for CHD aim to prevent the recurrence of MI or slow down the progression to heart failure; however, they do not promote cardiac repair. The advent of stem cell therapy introduced a viable approach for cardiac regeneration after MI.

Stem cell transplantation therapy offers the possibility for cellular regeneration of the heart after engraftment. Recently, stem cell therapy for cardiac regeneration has drawn intense attention with the discovery of induced-pluripotent stem cells (iPSC) (Takahashi and Yamanaka, 2006), which could provide an autologous source of cells to patients. Subsequent derivation of functional cardiomyocytes from iPSC (Zhang et al., 2009) offered the potential of direct regeneration of beating cardiac tissue. However, a critical barrier to stem cell therapy has been poor engraftment of the cells into the infarct area (Noort et al., 2012; van der Spoel et al., 2012). Another drawback of stem cell transplantation is the potential for teratoma formation (Cooke et al., 2006), even with highly purified cells (Fujikawa et al., 2005) and autologous grafting (Dlouhy et al., 2014). The fear of tumorigenicity has led to FDA guidelines dictating that cells must pass rigorous preclinical *in vivo* tumorigenicity testing in order to receive approval (Fox, 2008; Lee et al., 2013). Interestingly, modest improvements in cardiac function and increased angiogenesis have been observed with stem cell therapy despite poor survival or differentiation of the transplanted cells (den Haan et al., 2012; Noort et al., 2012; van der Spoel et al., 2012; Zuo et al., 2012; Bao et al., 2017; Wu et al., 2017). A growing body of evidence for the beneficial effect of stem cell therapy has pointed toward the paracrine factors of the transplanted cells. Further studies into this mechanism have identified EVs as a potent source of beneficial intercellular signaling. The significance of the paracrine signaling provided by these vesicles has been increasing since their discovery (Raposo et al., 1996).

In this study, the term EVs refers to two vesicle types shed by cells - exosomes and microvesicles. Additionally, the criteria to differentiate between exosomes and microvesicles includes their size and mechanism of biogenesis. Exosomes are small (50–150 nm) (Yanez-Mo et al., 2015) membrane-bound vesicles formed by an inward budding of multivesicular endosomes and subsequent fusion with the plasma membrane for secretion (Thery, 2011). Microvesicles bud directly from the plasma membrane and range in size from 100 to 500 nm (Colombo et al., 2014; Cocucci and Meldolesi, 2015). However, current techniques are not able to completely purify one type from the other and preparations should be collectively term EVs (Cocucci and Meldolesi, 2015; Sluijter et al., 2018). EVs have been found in numerous bodily fluids including blood and urine (Simons and Raposo, 2009; Mittelbrunn et al., 2011). They are capable of delivering active molecules to target cells including lipids (Record et al., 2014), protein (Choi et al., 2015), and nucleic acid (Gezer et al., 2014; Ahadi et al., 2016; Ohno and Kuroda, 2016). EV cargo is specific to their source cell type and its microenvironment (Thery et al., 2001; Mathivanan and Simpson, 2009). Thus, researchers are investigating how to optimize the paracrine signaling potential of EVs for use as a cell-free therapeutic. A major advantage of EVs is that they exhibit minimal tumorigenicity as they are readily taken up by cells or flushed out via the blood and urine as demonstrated by studies tracking the labeled exosomes (Lai et al., 2014, 2015). Additionally, EVs elicit minimal immune response as compared to stem cells (Bradley et al., 2002), which face the potential of rejection by the recipient. The relative safety of EVs makes them a compelling alternative to stem cell transplantation. Numerous studies have demonstrated the potential of EVs for stimulating angiogenesis, conferring cytoprotection, and modulating apoptosis.

Recent studies have demonstrated the beneficial role of EVs in improving cardiac function, angiogenesis, and decreasing fibrosis in treating animal models of MI. For example, exosomes secreted by mesenchymal stem cells (MSC) demonstrate their cardioprotective effects in rodent model of myocardial ischemia-reperfusion injury by decreasing infarct size and maintaining cardiac performance (Lai et al., 2010; Arslan et al., 2013; Bian et al., 2014) versus no exosome treatment. Similarly, exosomes from cardiosphere-derived cells (CDCs) decreased MI in mouse MI model, results exhibited decreased apoptosis and increased angiogenesis and cardiomyocyte proliferation. Additionally, blocking of exosome production by GW4869 abrogated the beneficial effects in the CDC conditioned media (Ibrahim et al., 2014). Mechanistic studies further determined that these exosomes were specifically enriched in miR-146a and experiments with miR-mimics showed that it was responsible for some of the beneficial effects conferred by the exosomes (Ibrahim et al., 2014). Exosomes isolated from cardiac progenitor cells (CPC) exhibit decreased cardiomyocyte apoptosis, increased angiogenesis, and improved cardiac function when injected into infarcted rat hearts (Barile et al., 2018). Recent research into exosomes derived from primary rodent cardiomyocytes (Garcia et al., 2015; Ribeiro-Rodrigues et al., 2017) and H9C2 cells (Ribeiro-Rodrigues et al., 2017) showed that exosomes harvested from cells subjected to glucose starvation (Garcia et al., 2015) or ischemia (Ribeiro-Rodrigues et al., 2017) stimulated angiogenesis. Our lab has previously demonstrated the beneficial effects of hiPSC-CM transplantation in improving cardiac function and attenuating fibrosis MI (Citro et al., 2014); similar to the findings from other labs (Zhang et al., 2015; Weinberger et al., 2016; Li et al., 2017) However, the role of EVs released by hiPSC-CM in promoting angiogenesis have not been explored yet. Therefore, the main objective of this study was to isolate, characterize, and evaluate the angiogenic potential of EVs secreted by hiPSC-CM *in vitro*.

## Materials and Methods

### Materials

Please see Supplementary Table 1 for a list of materials used in this work.

### Culture and Maintenance of hiPSC-CM and BAEC

Human induced pluripotent stem cell-derived cardiomyocytes were obtained from Cellular Dynamics Inc. (CDI, Appleton, WI, United States) and cultured as previously published (Citro et al., 2014; Khan et al., 2015b). Briefly, cells were plated onto gelatin-coated (0.1% gelatin, 1 h at 37°C) 6-well plates at 1 × 10^6^ viable cells per well in Cardiomyocyte Plating Medium (CDI, WI). After 48 h, media was changed to Cardiomyocyte Maintenance Medium (CDI, Appleton, WI, United States) and replenished every 2 days. BAEC were cultured as described previously (Dougherty et al., 2017a) in media containing endothelial cell growth supplement (ECGS) at 10 μg/ml, non-essential amino acids (NEAA), 10% FBS, and antibiotic/antimycotic. Cells were passaged and used sub-confluence, and cells from passages 5–9 were used for experiments.

### Analysis of hiPSC-CM for Expression of Cardiac Troponin T

Human induced pluripotent stem cell-derived cardiomyocytes were plated onto a 6-well plate at a density of 1.0 × 10^6^ cells/well. Cells were detached with trypsin and pelleted for analysis. The hiPSC-CM pellet was suspended in 250 μl of staining buffer and washed once with perm/wash buffer (BD Bioscience, San Jose, CA, United States). The hiPSC-CM pellet was suspended in cytofix/cytoperm buffer and incubated on ice for 30 min for fixation/permeabilization, then washed twice with perm/wash buffer. hiPSC-CM were incubated with either Alexa Fluor^®^ 647-conjugated mouse anti-cardiac troponin T (565744, BD Bioscience, San Jose, CA, United States) or Alexa Fluor^®^ 647-conjugated mouse IgG1 κ isotype control (557732, BD Bioscience, San Jose, CA, United States) on ice for 1 h. The stained hiPSC-CM were washed twice with BD perm/wash buffer. Finally, cells were resuspended in 400 μl of staining buffer and FACS acquisition was performed on BD^TM^ LSR II using BD FACSDiva^TM^ software (BD Bioscience, San Jose, CA, United States) while data analysis was performed using FlowJo^®^ version 10.4.1 (FlowJo, LLC; Becton, Dickinson and Company, Portland, OR, United States). For immunostaining, cells were fixed and incubated with Alexa Fluor^®^ 647-conjugated anti-cardiac troponin T (565744, BD Bioscience, San Jose, CA, United States) and NucBlue^®^ (Invitrogen, Carlsbad, CA, United States) was used to stain nuclei. Confocal microscopy was performed on an Olympus FV 3000 confocal microscope and the images were analyzed with Olympus FV31S-SW (Ver: 2.1.1.98).

### EVs Generation and Isolation

hiPSC-CM-EVs (CM-EVs) were collected from the cell supernatant at 2 weeks in culture. EV-generating media was prepared according to CDI’s protocol and supplemented with 10% exosome-depleted FBS (SBI, Mountain View, CA, United States). Medium was removed, cells were washed twice with basal DMEM (A14430, Life Technologies, Waltham, MA, United States), and EV media was added to cells. After 48 h, EV-containing media was collected and refreshed for another 48 h. Cell supernatant was collected at days 12–14 and 14–16 and pooled together for experimental use. After collection, media was clarified using a 0.22 μm syringe filter. EVs were precipitated by adding 1/5 volume of ExoQuick-TC reagent (SBI, Mountain View, CA, United States) to the media, inverting until homogenous, and incubating at 4°C overnight without disturbance. The media was centrifuged at 1,500 × *g* for 30 m at 4°C to pellet EVs. Supernatant was carefully removed and saved. Pellets were centrifuged again at 1,500 × *g* for 5 m at 4°C in order to remove residual supernatant. A second precipitation of the supernatant was performed by adding another 1/5 volume of ExoQuick-TC, inverting to mix, and incubating at 4°C overnight. EV pellets were suspended in PBS and stored at -80°C.

### Transmission Electron Microscopy (TEM) to Assess EV Size and Structure

Extracellular vesicles were fixed in 2% paraformaldehyde, loaded on 300-mesh formvar/carbon-coated electron microscopy grids (Electron Microscopy Sciences, Hatfield, PA, United States), post-fixed in 1% glutaraldehyde, and then contrasted and embedded as described previously^3^. TEM images are obtained with an FEI (Hillsboro, OR, United States) Tecnai Spirit G2 transmission electron microscope operating at 120 kV.

### Measurement of EV Size and Concentration by Nanoparticle Tracking Analysis (NTA)

Extracellular vesicles isolated from hiPSC-CM were diluted in 1 ml PBS and loaded onto a Malvern NanoSight NS300 (Malvern, United Kingdom) for NTA. Video captured on NanoSight camera was processed and analyzed using NanoSight NS300 NTA software v3.00 (Malvern, United Kingdom) to determine size distribution and particle concentration of the sample. Triplicate measurements were performed and averaged.

### Immunoblotting for EV Protein Markers

To estimate total EV protein, EVs were suspended in RIPA buffer (200 mM Tris-HCl (pH 7.4), 150 mM NaCl, 1% NP-40, 0.5% sodium deoxycholate, 0.1% SDS, 2 mM EDTA with protease and phosphatase inhibitors) and lysed on ice for 10 min. Protein estimation was performed with the Pierce BCA protein assay kit against a BSA standard curve. Lysed EVs were analyzed in triplicate and the values were averaged for the total protein estimate. For EV protein analysis, 25 μg of total EV protein was lysed as mentioned above. Protein was denatured in SDS loading buffer at 95°C for 10 m. Samples were separated on 4–15% SDS-PAGE and transferred to nitrocellulose membrane. Membranes were blocked with 5% milk in TBS-T (1X TBS with 0.1% Tween-20) for 1 h at RT, then probed with primary antibody at 1:1,000 with overnight incubation at 4°C with gentle rocking. Membranes were washed three times with TBS-T for 10 m each. Secondary antibody was added at 1:10,000 and incubated at 4°C for 2 h with gentle rocking. Membranes were washed three times with TBS-T and incubated with ECL^TM^ Western blotting detection reagents (GE Healthcare, United Kingdom) for 1 m at RT. Membranes were exposed to film and developed with a SRX-101A film processor (Konica Minolta, Japan).

### EVs Labeling and Uptake by Endothelial Cells

hiPSC-CM-EVs were labeled with PKH26 as previously described (Franzen et al., 2014) with a slight modification. Briefly, 90 μl (500 μg) of EVs was diluted in 250 μl of PBS. For labeling, 4 μl PKH 26 dye was added to 1 ml Diluent and mixed well by pipetting. Next, 250 μl of dye solution was added to EVs, mixed immediately and thoroughly, and incubated at RT for 5 m. 500 μl of 1% BSA in PBS was added to quench the reaction and incubated at RT for 1 m. EVs were pelleted by centrifugation at 100,000 × *g* for 70 m at 4°C. Supernatant was removed and pellet was washed with 1 ml PBS. Samples were centrifuged again at 100,000 × *g* for 70 m at 4°C, and pellet was suspended in 50 μl PBS for future experiments and analysis. To assess EVs uptake, BAEC were seeded onto gelatin-coated coverslips in a 24-well plate and allowed to attach overnight in media with exosome-depleted FBS. The next day, cells were washed and given fresh media and CM-EVs were added at a concentration of 100 μg/ml and incubated at 37°C, 5% CO_2_. After 2, 4, and 6 h cells were washed three times with PBS, fixed in 4% paraformaldehyde for 10 m at RT, and again washed with PBS. Cells were stained with ActinGreen^TM^ 488, a green fluorescent phalloidin that binds F-actin, and NucBlue^®^ according to manufacturer’s protocols. Confocal microscopy was performed to visualize the localization of labeled EVs in BAEC (A1Rsi Confocal Microscope, Nikon, Melville, NY, United States). Z-stack confocal imaging was performed on serial slices of BAEC labeled with CM-EVs at a higher magnification with Nikon FV1000 filter confocal microscope and image processing was accomplished using Imaris software (v.9.2.1, Bitplane).

### Wound Healing Assay for Assessing Cell Migration

BAECs were seeded in quadruplicate wells of a 24-well plate and grown to confluence. The cell monolayer was wounded with a 200 μl pipet tip, washed twice with PBS, and 250 μl of treatment media was added per well. Treatment media contained 1x DMEM basal media supplemented with 2% exosome-depleted FBS and CM-EVs at a concentration of 100 μg/ml, or an equal volume of PBS for control (no-EVs). Wells were imaged with EVOS^TM^ FL Auto 2 Imaging System (Thermo Fisher, Carlsbad, CA, United States) with four frames per well programmed into the scan protocol to capture the same fields over the course of the experiment. Images were captured at 10× magnification at 0, 10, and 24 h. Wound width was analyzed with ImageJ by a blinded operator drawing straight lines at three consistent points along the *y*-axis in all frames. Average length per well was calculated and percent wound closure was determined relative to time 0. Wound closure measurements were averaged from 4-wells in each group.

### Evaluation of Cell Proliferation by XTT Assay

BAECs were seeded into 96-well plates at 6,000 cells/100 μl per well in triplicate on separate plates for day 0, day 1, and day 2 measurements. Experiments were carried out with phenol red-free 1X DMEM basal media supplemented with 2% exosome-depleted FBS, ECGS, NEAA, and antibiotic/antimycotic. Cells were treated with EVs at 100 μg/ml or an equal volume of PBS for control (no-EVs). Media blanks of 100 μl were added to plates in triplicate. XTT cell proliferation assay (Cayman Chemical, Ann Arbor, MI, United States) was utilized per the manufacturer’s protocols. 10 μl of XTT reagent was added to each well, plates were shaken for 60 s at 500 rpm, and placed in 37°C cell incubator for 2 h. Plates were shaken for 1 m prior to measurement and the absorbance was measured at 450 nm for 1 s per well using a Victor^TM^ X3 2030 Multilabel Reader (Perkin Elmer, Waltham, MA, United States). Blank values were subtracted from sample values and fold change was calculated relative to the average of corresponding day 0 data.

### Tube Formation to Assess Angiogenesis *in vitro*

BAECs were seeded at 40,000 cells/250 μl per well in triplicate onto Growth Factor Reduced Geltrex^®^-coated wells (100 μl per well) of a 24-well plate in media containing basal DMEM with EVs at 100 μg/ml total EV protein (CM-EVs) or an equal volume of PBS (Control, no-EVs). Cells were incubated in a humidified 37°C 5% CO_2_ cell incubator for 16 h. Cells were then washed with PBS, fixed with 4% paraformaldehyde for 20 min at RT. Nine images per well were acquired at 10× magnification with a phase contrast microscope (Axio Vert.A1, Zeiss, Germany) and images were analyzed with the ImageJ Angiogenesis Analyzer macro, see Supplementary Figure 1 for settings. Well values were summed to get total-per-well values, and then averaged across three wells. Fold change in tube formation was calculated relative to the control (no-EVs). To assess the uptake of EVs in BAECs during tube formation, PKH26-labeled CM-EVs were added at a concentration of 100 μg/ml to BAEC for tube formation assay. After 16 h cells were washed, fixed, and stained with ActinGreen^TM^ 488 according to manufacturer’s protocols. Confocal laser microscopy was performed to visualize presence of labeled EV tubes using a fluorescence microscope (DM IL LED, Leica, Germany and A1Rsi Confocal Microscope, Nikon, Melville, NY, United States).

### qRT-PCR Profiling of Growth Factors in Endothelial Cells Treated With CM-EVs

Gene expression profiling for growth factors was performed in hiPSC-EC (human iPSC-derived endothelial cells) treated with CM-EVs. Briefly, hiPSC-EC were grown to 80% confluency. hiPSC-EC were treated with 100 μg/ml of CM-EVs, whereas and the other cell culture wells were treated with an equal volume of PBS (Control, no-EVs). After 48 h the media was removed, cells were washed three times with PBS, and 750 μl of Trizol was added to lyse the cells. Cells were scraped and placed in a 15 ml tube. Wells were rinsed with 750 μl 100% EtOH to harvest remnant cells and combined with the cells in Trizol. Total RNA was extracted using the Direct-zol RNA Miniprep kit (Zymo, Irvine, CA, United States) per the manufacturer’s protocol with on-column DNase digestion being performed. All of the cDNA used for gene expression analysis was prepared from the same master mix for the reverse transcription reactions to control for variability between preparations. First, cDNA was synthesized from 200 ng of total RNA with the RT^2^ First Stand Kit (Qiagen, Germantown, MD, United States) per the manufacturer’s protocol but with synthesis at 37°C for 1 h. Gene expression levels were analyzed with RT^2^ SYBR Green Master Mix on a custom RT^2^ Profiler PCR Array Plate with assays for growth factor mRNA as well as housekeeping genes (PGK0 and β-Actin), RT control, positive PCR control, and genomic DNA contamination control (See Supplementary Table 2 for assay details). Cycling for these reactions was as follows: 95°C 10 min → (95°C 15 s → 60°C 1 min^∗^) × 40 cycles, ^∗^ denotes fluorescent detection. For additional testing of VEGFA expression, 500 ng of total RNA was used to synthesize cDNA using the High Capacity cDNA Reverse Transcriptase Kit (Applied Biosystems, Foster City, CA, United States) per the manufacturer’s protocol. qRT-PCR was performed in triplicate wells with PowerUp SYBR Green Master Mix (Applied Biosystems, Foster City, CA, United States) with primers for VEGFA and housekeeping genes (RPL13a and β-Actin) (See Supplementary Table 3 for primer details). Cycling for these reactions was as follows: 95°C 10 min → (95°C 10 s → 60°C 10 s → 72°C 20 s ^∗^) × 40 cycles, ^∗^ denotes fluorescent detection. All qRT-PCR was performed on a QuantStudio 3 Real-Time PCR System (Thermo Fisher, Waltham, MA, United States). All samples were analyzed on the same array plate for the Qiagen assays (in duplicate) and simultaneously on the same 96-well plate for VEGFA analysis (in triplicate). Expression levels were calculated using the 2ˆ(-ΔΔCt) method (Livak and Schmittgen, 2001), with efficiency correction as required (Pfaffl, 2001), relative to Control (no-EVs) treatment with geometric normalization to two housekeeping genes (Vandesompele et al., 2002). HGF was also tested but not detected in any of the samples (Ct > 37) and thus is excluded from the graph. Data represents mean ± SD, *n* = 3 (Biological triplicates).

### Statistical Analysis

Statistical analysis was performed in SigmaPlot 13 software (Systat Software, Inc.) using one-way ANOVA with Tukey’s post-test at alpha = 0.05, a value of *p* < 0.05 was considered statistically significant. All values are expressed as mean ± SD.

## Results

### Evaluation of hiPSC-CM Cardiac Phenotype by Flow Cytometry

**FIGURE 1:**
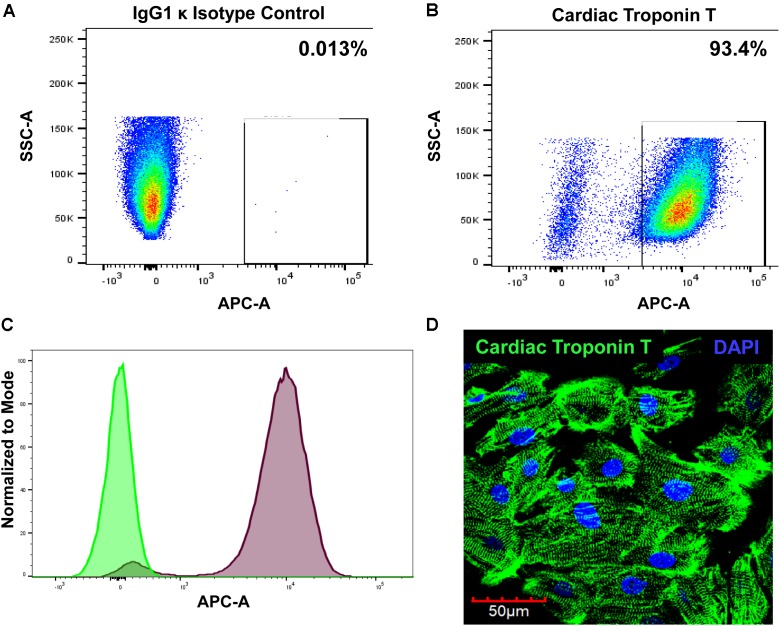
Evaluation of cardiomyocyte phenotype. The hiPSC-CMs were cultured for 2 weeks and then processed for analysis. The cells were stained with either **(A)** Alexa Fluor^®^ 647-conjugated IgG1 κ isotype control or **(B)** Alexa Fluor^®^ 647-conjugated cardiac troponin T and analyzed with FACS. **(C)** Overlay histogram of IgG1 κ isotype control (green) and cardiac troponin T signal (red). **(D)** Immunostaining of hiPSC-CM with cardiac troponin T.

Cardiomyocytes express a cardiac-specific isoform of troponin T, TNNT2 or cardiac troponin T (Wilhelm et al., 2014). To confirm cardiomyocyte phenotype of hiPSC-CM, cells were assessed for cardiac troponin T expression with flow cytometry and immunostaining. FACS analysis shows that 93.4% of hiPSC-CMs are positive for cardiac troponin T (Figures 1A–C). Immunofluorescent staining of cells further demonstrated strong expression of cardiac troponin T and well-defined and prominent sarcomeric organization in cardiomyocytes (Figure 1D), as seen in cardiac tissue.

### Transmission Electron Microscopy (TEM) and Nanoparticle Tracking Analysis (NTA) to Assess the Structure, Size, Distribution, and Concentration of EVs Released by hiPSC-CMs

**FIGURE 2:**
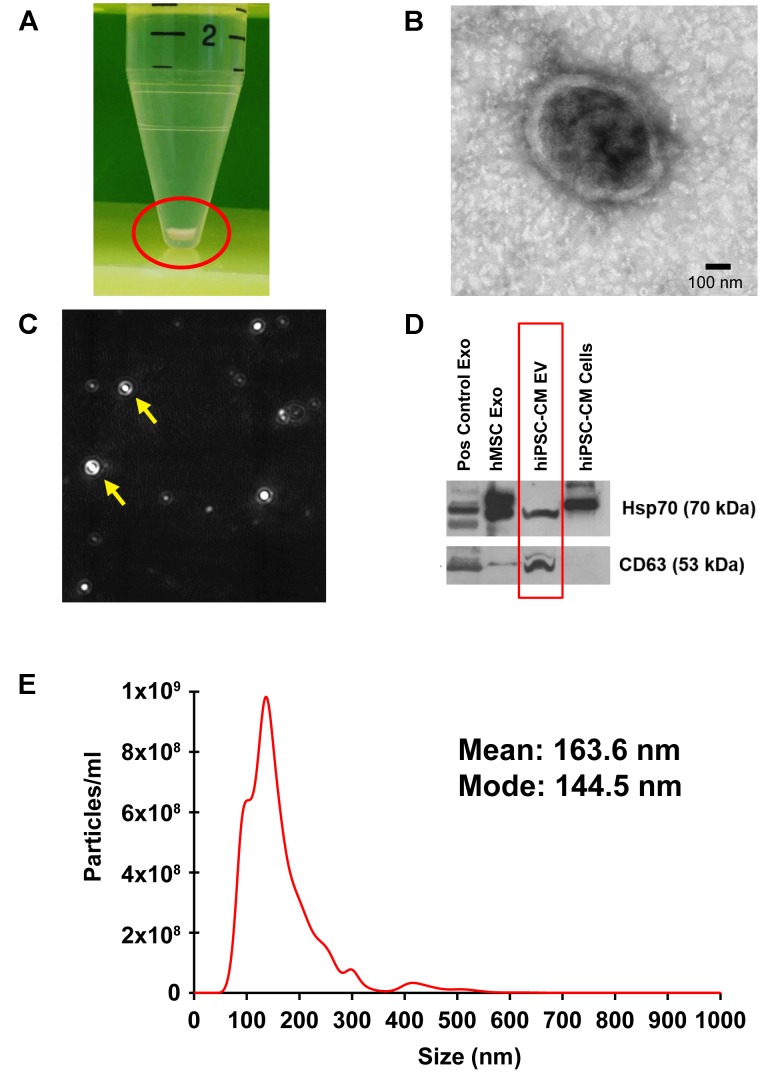
Localization, isolation, and analysis of EVs from hiPSC-CM. **(A)** EVs were isolated from the culture medium of hiPSC-CM using ExoQuick-TC and pelleted with centrifugation. **(B)** TEM of isolated EVs. **(C)** A camera view of NTA showing EVs moving in Brownian motion during analysis. **(D)** Protein from EVs and cell lysates were analyzed by immunoblotting for exosome-enriched proteins, Hsp70 (upper panel) and CD63 (lower panel). Positive control human exosome lysate (Pos control Exo), exosomes isolated from human-MSCs (hMSC Exo), and hiPSC-CM-EVs express exosome markers. Protein from hiPSC-CM cell lysate served as control. **(E)** Size distribution of hiPSC-CM-derived EVs analyzed by NTA in triplicate.

Transmission electron microscopy analysis performed on the isolated EVs confirmed the presence of double membrane-bound vesicle structure (Figure 2B). Furthermore, NTA analysis measured the particle number and size as particles are detected by the light scattered when irradiated with a laser (Figure 2C). Size measurement is based on Brownian motion, where the diffusion of particles in a liquid is inversely proportional to size (Mehdiani et al., 2015). EVs isolated from hiPSC-CM-conditioned medium (Figure 2A) were analyzed for size, distribution, and concentration with NTA on a Malvern NanoSight NS300 (Figure 2E). CM-EVs had a mean particle size of 163.6 nm and a mode particle size of 144.5 nm (Figure 2E). These sizes correlated with the characteristic size of exosomes (Yanez-Mo et al., 2015) and microvesicles (Colombo et al., 2014). Particle concentration was determined to be 9.95 × 10^10^ particles/ml. These results are further evidence that hiPSC-CMs secrete EVs.

### Western Blot Analysis to Detect EV Surface Markers

Proteomic analyses show that exosomes have a protein signature highly enriched in tetraspanins, including CD63 (Andreu and Yanez-Mo, 2014; Schey et al., 2015). Additionally, enrichment of heat shock proteins in exosomes, including Hsp70, has been demonstrated by numerous studies (Lancaster and Febbraio, 2005; Zhan et al., 2009; De Maio and Vazquez, 2013). hiPSC-CM-EV protein was analyzed with immunoblotting using antibodies to CD63 and Hsp70. MSC-derived exosomes and company provided positive control exosomes, and hiPSC-CM cell lysate were included for comparison. The upper panel positively detects Hsp70 in all exosome samples and the cell lysate (Figure 2D). The lower panel demonstrates detection of CD63 in all EV samples (Figure 2D).

### Internalization of PKH26 Labeled CM-EVs in BAECs

**FIGURE 3:**
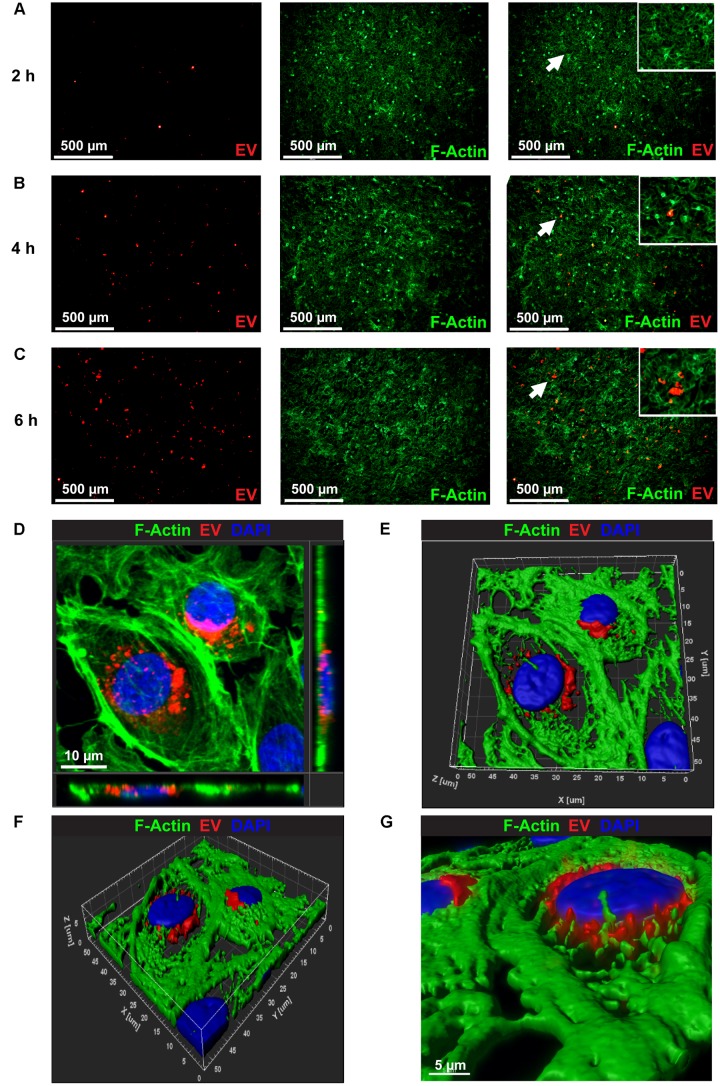
Confocal microscopy images showing the uptake of PKH26 labeled EVs by BEAC *in vitro*. Time-dependent uptake of PKH26-labeled EVs were visualized in BAEC at **(A)** 2 h, **(B)** 4 h, and **(C)** 6 h of incubation. **(D)** Representative Z-stack confocal image through the volume of the treated cells that demonstrates the internalization and localization of PKH26-labeled EVs (red color) to the perinuclear region of BAEC (F-actin, green color). **(E,F)** A volume rendered 3-D reconstruction of the fluorescent Z-stack image **(D)** in different orientations, which further demonstrates the localization of EVs in perinuclear region of BAEC. **(G)** Zoomed view of the 3-D reconstructed fluorescence image **(F)**.

To examine uptake of hiPSC-CM-derived exosomes by endothelial cells, 100 μg/ml of PKH26-labeled EVs were incubated with BAEC. At each time-point, (2, 4, and 6 h) cells were washed to remove unbound exosomes and fixed with paraformaldehyde. PKH26-labeled EVs (Red) were visible in BAEC as early as 2 h (Figure 3A). EVs uptake increased over time as evidenced by increased red fluorescence at 4 h and 6 h (Figures 3B,C). To acquire a Z-stack confocal image, several serial slices were acquired from BAECs labeled with CM-EVs at a higher magnification to demonstrate the internalization and localization of labeled EVs in the cytoplasm and perinuclear regions of BAEC (Figure 3D). Volume rendered 3-D reconstructed fluorescent images further confirm localization of EVs in the cytoplasm and perinuclear region of BAECs (Figures 3E–G). These data demonstrate hiPSC-CM-EVs are readily taken up by endothelial cells.

### Functional Analysis to Assess the Angiogenic Potential of CM-EVs

Angiogenesis is the process of forming new blood vessels from existing ones (Adair and Montani, 2010). There are many steps to the process including cell migration, proliferation, and tube formation. We analyzed the angiogenic potential of CM-EVs with functional assays to measure their effect on cell migration, proliferation, and tube formation. We utilized BAECs as reporters for these assays since our data shows that hiPSC-CM-EVs are readily taken up by BAECs and they are a well-documented endothelial cell line in use in our lab (Barajas-Espinosa et al., 2014, 2015; Dougherty et al., 2017a).

#### (a) CM-EVs Promote Endothelial Cell Migration

**FIGURE 4:**
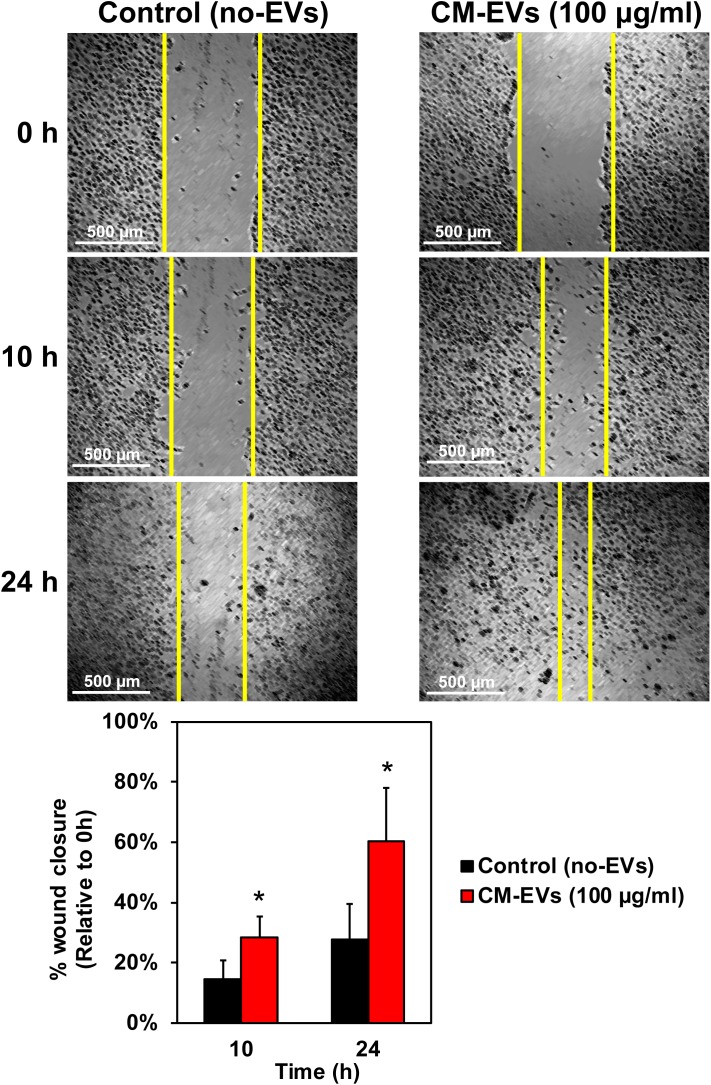
Effect of hiPSC-CM-EVs on cell migration. BAEC were seeded to 24-well plates and grown to confluence. Cells were wounded with 200 μl tip, washed gently, and the same four frames per well were imaged at 0 h, 10 h, and 24 h post-scratch. Wound width was measured by a blinded operator. Average width per well was calculated and % wound closure was determined relative to 0 h. Data represents as mean ± SD, *n* = 4, ^∗^*p* < 0.05.

Cell migration is a key step in the early phase of angiogenesis, cells must migrate toward the formation site of the new vessel (Adair and Montani, 2010). The wound-healing scratch assay is a well-established assay for 2-D cell migration (Rodriguez et al., 2005). BAECs were grown to confluence in 24-well plates and wounded with a 200 μl pipet tip. After washing, cells were incubated with treatment media and imaged at 10× magnification. Results demonstrate that treating BAECs with hiPSC-CM-derived EVs during the scratch assay resulted in increased wound closure over time (Figure 4). After 10 h, the control (no-EVs) wounds were only 14.7% ± 6.1% closed whereas the CM-EVs-treated were 28.5% ± 6.9% closed (Figure 4) (*p* < 0.01, *n* = 4). At 24 h, the percentage closure for CM-EVs treated cells was significantly increased when compared to control (no-EVs). Furthermore, control cells (no-EVs) showed only 27.7% ± 11.7% wound closure while CM-EVs treated cells exhibited 60.5% ± 17.6% closure (*p* < 0.05, *n* = 4) (Figure 4). These results demonstrate that EVs isolated from hiPSC-CMs promote migration of endothelial cells, a fundamental step in angiogenesis.

#### (b) CM-EVs Enhance Endothelial Cell Proliferation

**FIGURE 5:**
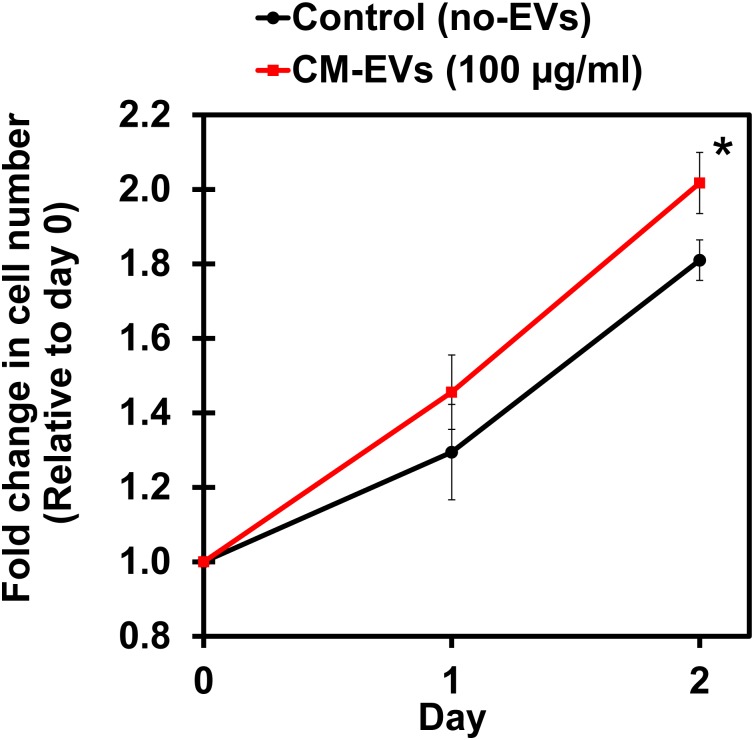
Effect of hiPSC-CM-EVs on cell proliferation. BAEC were seeded in triplicate in 96-well plates in EC growth media supplemented with 2% exosome-depleted FBS and treated with 100 μg/ml of CM-EVs (red line) or an equal volume of PBS for control (no-EVs; black line). XTT assay was used to measure cell number on days 0, 1, and 2. Fold change in cell number was calculated relative to day 0. Data represents as mean ± SD, *n* = 3, ^∗^*p* < 0.05.

Cell proliferation is an important aspect of angiogenesis as more cells are needed to form the new vessels (Adair and Montani, 2010). XTT, a tetrazolium salt-based assay, was used to measure the increase in cell number (Berridge et al., 2005). Cells were assayed with XTT on days 0, 1, and 2 and the blank-corrected absorbance was compared relative to day 0. After 2 days, BAEC treated with CM-EVs had significantly (*p* < 0.05, *n* = 3) increased cell number (2.02 ± 0.08) as compared to control (no-EVs) (1.81 ± 0.05) (Figure 5). Therefore, CM-EVs stimulate endothelial cell proliferation, which is important for angiogenesis.

#### (c) hiPSC-CM-EVs Promote Angiogenesis in BAEC *in vitro*

**FIGURE 6:**
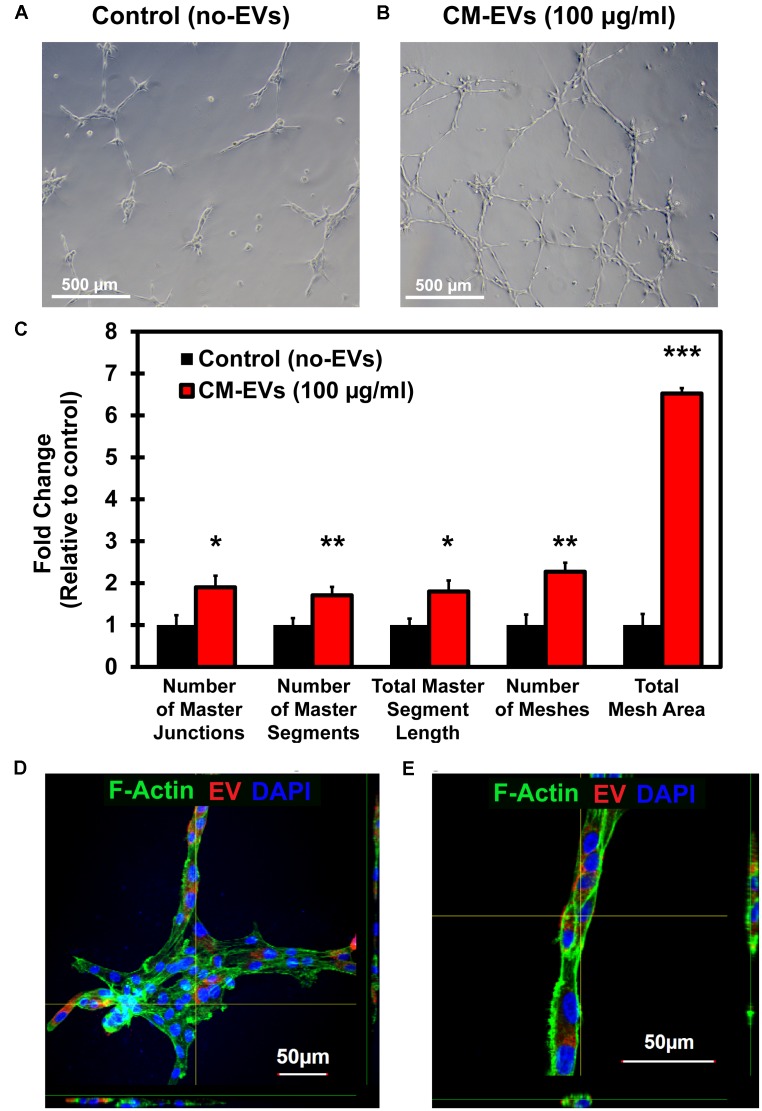
Tube formation potential of hiPSC-CM-EVs. Tube formation assays were conducted with BAECs seeded onto Geltrex^®^matrix and treated with 100 μg/ml hiPSC-CM EVs or an equal volume of PBS. After 16 h, cells were imaged at 10× magnification and analyzed with ImageJ Angiogenesis Analyzer macro. Representative phase-contrast images for **(A)** control (no-EVs) and **(B)** CM-EVs treated cells. **(C)** EVs treatment increased the number of master junctions, increased the number of master segments, increased total master segment length, increased number of meshes, and increased the total mesh area. Data was calculated as fold change relative to control (no-EVs). Data represents as mean ± SD, *n* = 3, ^∗^*p* < 0.05, ^∗∗^*p* < 0.01, ^∗∗∗^*p* < 0.0001. **(D)** Confocal imaging (Z-stack) of BAEC internalized with CM-EVs labeled with PKH26 (red fluorescent cell tracker) at 16 h cells after the treatment. The fluorescent CM-EVs was visualized in the nodes and tubes **(D,E)** during tube formation assay.

Reorganization of endothelial cells to form tubules is vital to new vessel formation to allow for subsequent blood flow (Adair and Montani, 2010). The tube formation assay using endothelial cells is a well-characterized model system for *in vitro* angiogenesis (Arnaoutova et al., 2009). BAEC were seeded with media containing 100 μg/ml EVs or an equivalent volume of PBS. After 16 h, cells were imaged at 10× magnification (Figures 6A,B) and images were analyzed by the ImageJ Angiogenesis Analyzer macro (Supplementary Figure 1). Fold change in tube formation was calculated relative to control (no-EVs) (Figure 6C). The number of master junctions increased significantly (*p* < 0.05) with CM-EVs treatment (1.90 ± 0.28) versus control (no-EVs) (1.00 ± 0.23). The number of master segments also increased significantly (*p* < 0.01, *n* = 3) with CM-EVs (1.71 ± 0.20) treatment versus control (no-EVs) (1.00 ± 0.16). The total length of master segments increased significantly (*p* < 0.05, *n* = 3) with CM-EVs (1.80 ± 0.26) treatment versus control (no-EVs) (1.00 ± 0.15). The total number of meshes also increased significantly (*p* < 0.01, *n* = 3) with CM-EVs (2.27 ± 0.21) treatment versus control (no-EVs) (1.00 ± 0.25). The total mesh area increased significantly (*p* < 0.0001, *n* = 3) with CM-EVs (6.52 ± 0.13) treatment versus control (no-EVs) (1.00 ± 0.26). Furthermore, uptake of labeled EVs during tube formation was also investigated. After 16 h of incubation, CM-EVs are readily seen inside BAECs forming nodes and tubes on Geltrex^®^-coated plates (Figures 6D,E). Thus, EVs derived from hiPSC-CMs promote endothelial cell tube formation, an essential feature of angiogenesis.

### CM-EVs Enhance Growth Factor Expression in Endothelial Cells

**FIGURE 7:**
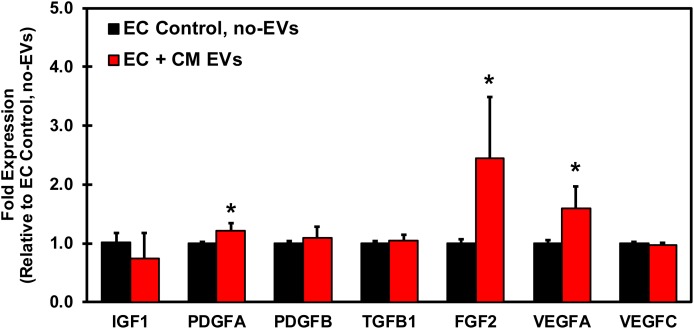
qRT-PCR Profiling of growth factors in endothelial cells treated with CM-EVs. hiPSC-EC were incubated for 48 h with CM-EVs or PBS (Control, no-EVs). Expression of growth factors involved in angiogenesis were assessed by qRT-PCR and calculated relative to ECs without EV treatment after geometric normalization to housekeeping genes, shown as mean ± SD, *n* = 3, ^∗^*p* < 0.05.

In order to understand the underlying mechanism by which CM-EVs promote angiogenesis in endothelial cells, hiPSC-EC were incubated with CM-EVs for 48 h and gene expression levels of growth factors in endothelial cells were analyzed by qRT-PCR. Several important pro-angiogenic growth factors demonstrated a significant increase in expression with CM-EV treatment. FGF expression significantly (*p* < 0.05, *n* = 3) increased (2.45 ± 1.05) (Figure 7). Similarly, VEGFA expression also increased significantly (*p* < 0.05, *n* = 3) (1.59 ± 0.37), as compared to control (1.006 ± 0.048) (Figure 7). Furthermore, expression of PDGFA significantly (*p* < 0.05, *n* = 3) increased (1.21 ± 0.13), as compared to control cells (1.00 ± 0.03) (Figure 7). Overall, these results suggest that CM-EVs promote angiogenesis in part by inducing an increased expression of pro-angiogenic growth factors in endothelial cells.

## Discussion

One of the major advantages of using hiPSC-CM lies in their potential for autologous use in patients for myocardial regeneration and for disease modeling. Human iPSC-CM transplanted post-MI have demonstrated improved cardiac function and attenuated fibrosis in rat models (Miki et al., 2012; Citro et al., 2014; Zhao et al., 2018). However, there is a paucity of information about EVs released by these terminally differentiated cardiomyocytes. Our results demonstrated that hiPSC-CM-derived EVs promote increased angiogenesis in endothelial cells *in vitro*. EVs released from hiPSC-CMs could serve as novel cell-free therapeutics for patients with MI to promote angiogenesis in the infarct region. EVs secreted by cells can be classified as apoptotic bodies, microvesicles, or exosomes based on their origin and size (Raposo and Stoorvogel, 2013). TEM imaging illustrated the size and structure of nanoparticles secreted by hiPSC-CM to be congruent with that characteristic of EVs (Colombo et al., 2014; Yanez-Mo et al., 2015). NTA data further demonstrated that EVs isolated from hiPSC-CM had a mode size of 144.5 nm, and mean size of 163.6 nm. Proteomics studies have identified several proteins enriched in exosomes, including CD63 and Hsp70 (Thery et al., 1999, 2001; Subra et al., 2010). Both of these exosome markers were detected in hiPSC-CM-EVs by immunoblotting, further confirming their identity as EVs. The uptake of EVs by target cells can occur via many pathways (Dougherty et al., 2017b), varies by cell type (Svensson et al., 2013), and is dose-dependent (Franzen et al., 2014). Importantly, the hiPSC-CM-derived EVs were easily internalized in endothelial cells under normal culture conditions and during tube formation assay. We then investigated whether these EVs could have functional effects on endothelial cells.

Angiogenesis is a multistep process involving cell migration, proliferation, and tube formation. EVs isolated from numerous cell types have demonstrated proangiogenic effects. Cell migration and proliferation are key facets of angiogenesis, which could be stimulated by EVs. MSC-derived exosomes/EVs promote migration of fibroblasts (Shabbir et al., 2015; Ferreira et al., 2017), keratinocytes (Ferreira et al., 2017), and endothelial cells (Vrijsen et al., 2010). Moreover, exosomes isolated from CPCs promote endothelial cell migration (Vrijsen et al., 2010). A study of human pericardial fluid-derived exosomes revealed they stimulated proliferation of endothelial cells (Beltrami et al., 2017). Furthermore, exosomes derived from mouse embryonic stem cells stimulated proliferation of resident CPCs when injected into infarct mouse hearts (Khan et al., 2015a) and cardiosphere-derived exosomes stimulated proliferation of cardiomyocytes in the peri-infarct region of pig hearts (Gallet et al., 2017). Similarly, this study demonstrated that hiPSC-CM-derived EVs significantly promoted endothelial cell migration and proliferation.

The hallmark aspect of angiogenesis is tube formation, which allows blood to flow through the new vessel. Studies using multiple cells types have demonstrated their derived EVs enhance tube formation. A study of exosomes isolated from CDCs showed increased angiogenesis in tube formation assays and increased density of microvessels when delivered to infarcted mouse hearts (Ibrahim et al., 2014). A pig model of MI also exhibited increased vessel density in the infarcted heart after treatment with CPC-derived exosomes (Gallet et al., 2017). Additionally, a study by Sahoo et al. (2011) utilized exosomes derived from human CD34+ stem cells and observed increased *in vitro* tube formation and increased *in vivo* vessel formation with Matrigel plug assay. A study by Vrijsen et al. (2016) used exosomes isolated from CPCs and MSCs and showed increased *in vitro* tube formation and increased sprouting. Further experiments identified EMMPRIN, VEGF, and MMP-9 within the exosomes and determined EMMPRIN to be the major proangiogenic factor (Vrijsen et al., 2016). Salomon et al. (2013) demonstrated that exosomes isolated from placental MSCs increased tube formation *in vitro*. LC-MS/MS and Ingenuity Pathway Analysis identified VEGF-signaling and actin cytoskeleton signaling as the major pathways induced by exosomes treatment (Salomon et al., 2013). Another study using exosomes isolated from human umbilical cord blood showed increased tube formation *in vitro* with exosomes treatment (Hu et al., 2018). Mechanistic studies determined miR-21-3p to be the major effector causing down-regulation of PTEN and SPRY1 and increased phosphorylation of Akt and ERK1/2 (Hu et al., 2018). Likewise, our results demonstrated that treatment of BAECs with hiPSC-CM-derived EVs enhances *in vitro* angiogenesis by significantly increasing several facets of tube formation.

Angiogenesis is a complex process involving the coordination of numerous growth factors, with major players being VEGFA and FGF2. Recent studies have shown that FGF2 is a more potent activator of angiogenesis than VEGFA (Seghezzi et al., 1998) and is regarded as a leading potential therapeutic for inducing angiogenesis in the ischemic heart (Felmeden et al., 2003). Additionally, PDGFA has also demonstrated pro-angiogenic effects (Sahraei et al., 2012; Palomero et al., 2014) and the PDGF family is structurally and functionally related to the VEGF family of growth factors (Andrae et al., 2008). Overall, the results from our study demonstrated increased gene expression of FGF2, VEGFA, and PDGFA in endothelial cells, thus indicating a possible mechanism of action of CM-EVs for promoting angiogenesis *in vitro*.

## Conclusion

In summary, this study demonstrates that EVs secreted by human iPSC-CMs stimulate *in vitro* angiogenesis, along with increased expression of FGF2, VEGF2A, and PDGFA in endothelial cells. The significance of our findings could have meaningful clinical impact, as patient-derived hiPSC-CM could be used to generate autologous EVs that could be administered therapeutically post-MI to promote angiogenesis. The use of a terminally differentiated cell type greatly decreases the risk of tumorigenicity and autologous therapies confer minimal immunologic response from patients. Furthermore, the use of EVs as a cell-free therapeutic may serve as a potential viable option for enhancing angiogenesis in patients after MI.

## Author Contributions

JD and MaK: conceptualization. JD, NK, C-AC, MoK, and MaK: methodology. JD, NK, MN, C-AC, and MaK: data analysis. MoK: TEM analysis. JD, C-AC, and MaK: writing-manuscript draft. JD, C-AC, MA, and MaK: reviewing and editing. MA and MaK: funding. C-AC and MaK: supervision. MaK guided and directed all the work. JD and MaK prepared the final manuscript with input from all the authors. All authors discussed the results and commented on the manuscript.

## Conflict of Interest Statement

The authors declare that the research was conducted in the absence of any commercial or financial relationships that could be construed as a potential conflict of interest.

## Abbreviations

BAEC: bovine aortic endothelial cells
CHD: coronary heart disease
CM-EVs: human induced-pluripotent stem cell-derived cardiomyocytes extracellular vesicles
CVD: cardiovascular disease
EC: hiPSC-derived EC
EVs: extracellular vesicles
FACS: fluorescence-activated cell sorting
FGF: fibroblast growth factor
hiPSC: human induced-pluripotent stem cells
hiPSC-CM: human induced-pluripotent stem cell-derived cardiomyocytes
hiPSC-EC: hiPSC-derived endothelial cells
MI: myocardial infarction
NTA: nanoparticle tracking analysis
PDGF: platelet-derived growth factor
TEM: transmission electron microscopy
VEGF: vascular endothelial growth factor
