# Supplementary material for: Extracellular Vesicles Released by Human Induced-Pluripotent Stem Cell-Derived Cardiomyocytes Promote Angiogenesis
Source: Front Physiol. 2018 Dec 14;9:1794. doi: 10.3389/fphys.2018.01794 (PMC6302004; doi:10.3389/fphys.2018.01794)
Supplement: Supplementary file 1 [file Data_Sheet_1.PDF]

Supplementary Table 1. List of materials used in the experiments.

Reagent	Catalog #	Company
iCell® Cardiomyocytes	CMC-100-010-001	Cellular Dynamics International, Inc
iCell® Cardiomyocytes Maintenance Media	CMM-100-120-005	Cellular Dynamics International, Inc
iCell® Cardiomyocytes Plating Media	CMM-100-110-005	Cellular Dynamics International, Inc
ActinGreen™488	R37110	Invitrogen™(Thermo Fisher Sci)
NucBlue®	R37606	Invitrogen™(Thermo Fisher Sci)
Exosome-depleted FBS	EXO-FBS-250A-1	System Biosciences
DMEM 1X (hiPSC-CM)	A14430	Life Technologies
PKH26 Red Fluorescent Cell Linker	MINI26-1KT	Sigma Aldrich
Hsp70 primary Antibody	EXOAB-Hsp70A-1	System Biosciences
CD63 primary antibody	EXOAB-CD63A-1	System Biosciences
Human Exosome lysate positive protein control	EXOAB-POS-1	System Biosciences
Alexa Fluor® 647 mouse anti-cardiac troponin T	565744	BD Bioscience
Alexa Fluor® 647 mouse IgG1 κ isotype control	557732	BD Bioscience
Endothelial Cell Growth Supplement	02-102	Millipore
Non-Essential Amino Acids (NEAA) 100X	11140-020	ThermoFisher Scientific
Geltrex® Reduced Growth Factor	A1413202	ThermoFisher Scientific
Endothelial Cell Growth Supplement	02-102	Millipore
DMEM 1X (BAEC)	11885-084	ThermoFisher Scientific
DMEM 1X, phenol red free (BAEC)	11054-020	ThermoFisher Scientific
Antibiotic/antimycotic 100X	15240-062	ThermoFisher Scientific
XTT cell proliferation assay	10010200	Cayman Chemical
TRIzol™ Reagent	15596018	Thermofisher Scientific
Direct-zol RNA Miniprep	R2050	Zymo Research
RT^2 First Strand Kit	330401	Qiagen
RT^2 SYBR Green Master Mix	330523	Qiagen
High Capacity cDNA RT Kit	4368814	Applied Biosystems
PowerUp™ SYBR© Green Master Mix	A25742	Applied Biosystems

Supplementary Table 2. Qiagen RT2 Profiler PCR Array details.

Gene/Assay	Array #
IGF1	PPH00167C
PDGFA	PPH00217C
PDGFB	PPH00488F
TGFB1	PPH00508A
HGF	PPH00163C
FGF2	PPH00257C
VEGFC	PPH00673D
PGK1 (HKG)	PPH02049A
ACTB (HKG)	PPH00073G
RTC (RT control)	PPX63340
PPC (Positive PCR control)	PPX63339
GDC (gDNA control)	PPH65835A

Supplementary Table 3. qRT-PCR primer details.

Primer	Sequence	Primer Efficiency	Final Conc	Product Length
VEGFA F	TCCACCATGCCAAGTGGTC	105.33%	500 nM each	99
VEGFA R	AGCTGCGCTGATAGACATCC			
RPL13A F	GCCTACAAGAAAGTTTGCCTAT	94.69%	500 nM each	123
RPL13A R	CTTCTTCCGGTAGTGGATCTT			
BACT F	CTGGAACGGTGAAGGTGACA	93.75%	500 nM each	139
BACT R	AAGGGACTTCCTGTAACAATGCA			

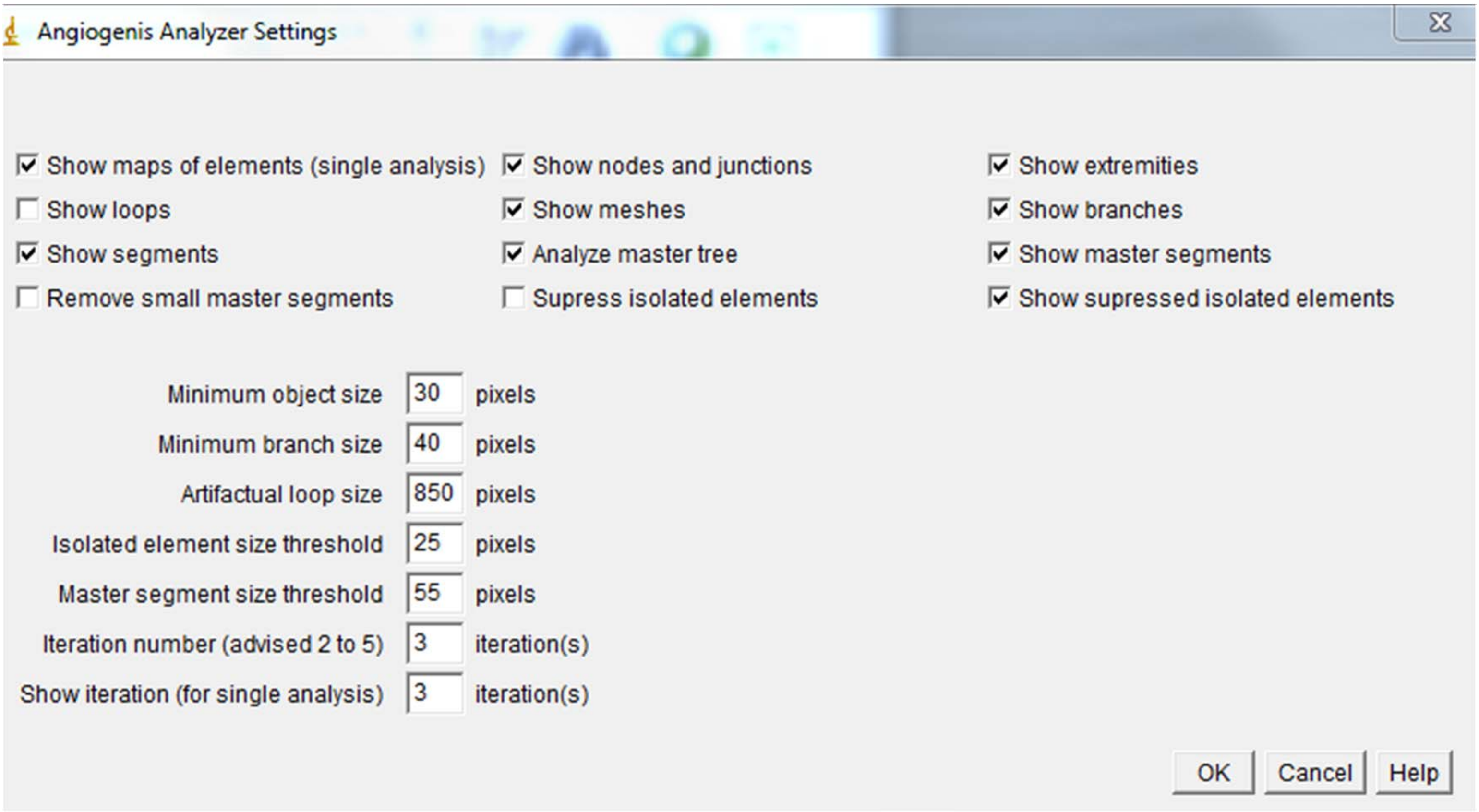

Supplementary Figure 1. Settings for Angiogenesis Analyzer analysis of phase contrast images. Values were adjusted from defaults to correctly identify elements in images. Image size was 2463x2056 pixels in tiff format.
